# Impact of three genetic musculoskeletal diseases: a comparative synthesis of achondroplasia, Duchenne muscular dystrophy and osteogenesis imperfecta

**DOI:** 10.1186/s12955-014-0151-y

**Published:** 2014-10-25

**Authors:** Maman Joyce Dogba, Frank Rauch, Erin Douglas, Christophe Bedos

**Affiliations:** Shriners Hospital for Children, 1529 Cedar Avenue, H3G 1A6 Montreal, QC Canada; Department of family and emergency medicine, Faculty of Medicine, Université Laval, 1050 Medicine Avenue, Quebec, G1V0A6 Canada; Faculty of Dentistry, McGill University, 3550 University Street, H3A 2A7 Montreal, QC Canada; Department of Social and Preventive Medicine, Faculty of Medicine, Université de Montréal, H3C 3 J7, C.P. 6128, Succ. Centre-Ville, Montreal, QC Canada

**Keywords:** Rare genetic diseases, Societal and family impact, Burden of care, Achondroplasia, Duchenne muscular dystrophy, Osteogenesis imperfecta, Quality of life, Scoping review

## Abstract

Achondroplasia, Duchenne muscular dystrophy, and osteogenesis imperfecta are among the most frequent rare genetic disorders affecting the musculoskeletal system in children. Rare genetic disorders are severely disabling and can have substantial impacts on families, children, and on healthcare systems. This literature review aims to classify, summarize and compare these non-medical impacts of achondroplasia, Duchenne muscular dystrophy and osteogenesis imperfecta.

## French abstract

L’achondroplasie, la dystrophie musculaire de Duchenne et l’ostéogenèse imparfaite sont des maladies génétiques rares du système musculo-squelettique pédiatrique. On assiste à un intérêt croissant pour l’étude des maladies génétiques rares dont la plupart sont handicapantes et ont des conséquences néfastes sur familles, les patients et les systèmes de santé. Malgré leurs spécificités, certaines conséquences des maladies génétiques rares sont communes et gagneraient à être étudiées de façon générique. Cependant, plusieurs travaux continuent d’adopter une approche spécifique à une maladie. Par ailleurs, on assiste à un foisonnement de termes qui évaluent les conséquences des maladies génétiques rares (qualité de vie, impact, effet, fardeau etc.…). Si ce pléthore de termes reflète la complexité du sujet, il rend difficile la synthèse et la comparaison entre maladies. Dans cet article, les auteurs classifient puis effectuent une revue narrative et comparative des impacts de trois maladies génétiques musculo-squelettiques pédiatriques: l’achondroplasie, la dystrophie musculaire de Duchenne et l’ostéogenèse imparfaite.

## Introduction

A rare disease is defined as one that affects less than one in 2000 individuals in Europe and one in 1250 in the United States [1]. Most rare diseases are severely disabling genetic disorders that can have substantial impacts on families and children, and on healthcare systems [2,3]. Despite their specific biomedical features, many rare genetic diseases (RGDs) share several non-medical characteristics, in particular, their psychosocial consequences [4]. While it is acknowledged that research on these non-medical common features may benefit from a non-categorical approach, a disease-specific approach remains common. In addition, the literature assessing these issues is sometimes difficult to summarize due to the inconsistent use of terminology. Several terms are commonly used in the literature to examine the impacts of RGDs: burden of care, quality of life, impacts, consequences, meaning of leaving, and coping strategies [5-7]. This proliferation of concepts, which depends on research interest and disciplinary tradition (e.g., biomedical, psychological, economic, social and nursing) reflects the complexity of the field, but may also lead to a fragmented version of ‘the same reality’. A clear and synthetized conceptualization of the impacts of RGDs with a non-disease-specific approach is warranted. Scoping reviews, an increasingly popular knowledge synthesis approach in health care, can help in such conceptualization [8]. Moreover, scoping reviews can yield a framework for collating and summarizing results that can empower patients and families, raise awareness among health care professionals, identify knowledge gaps and priorities for future research, and advocate for policies to develop support services for families [8,9].

This paper reports a scoping review that describes the literature on non-medical impacts for patients and families. Due to the exploratory nature of the review and to reflect the research areas of our team, we delineated the focus of this review to three common RGDs in the pediatric orthopedic context: achondroplasia, Duchenne muscular dystrophy (DMD) and osteogenesis imperfecta (OI). These three RGDs are single-gene musculoskeletal diseases characterized by physical disability and little or no impairment of mental ability. Table 1 provides their clinical and genetic characteristics.

**Table 1:** **Key features of achondroplasia, DMD and OI**

**Disease, features**	**Achondroplasia**	**Duchenne muscular dystrophy**	**Osteogenesis imperfecta**
Orphanet number and synonyms	ORPHA 15	ORPHA 98896	ORPHA 666
Prevalence	4/100,000	30/100,000 males	10/100,000
Genetic defect	Change in the DNA for fibroblast growth factor receptor 3 (FGFR3), which causes an abnormality of cartilage formation	Absence of the protein dystrophin in skeletal muscle, myocardium, and brain	Mutation in gene encoding collagen type I; deficiencies in proteins that interact with collagen (CRTAP, P3H1, PPIB, Serpin H1 and FKBP10)
Mode of transmission	Autosomal dominant	X-linked recessive	Primarily autosomal dominant, although recessive cases reported
Clinical symptoms	Short stature	Progressive weakness, contractures, spinal deformity, restrictive lung functional pattern, and cardiomyopathy	Increased bone fragility; fractures
Cognitive impairment	None noted	Some variable degree of cognitive involvement	None noted
Diagnosis	Neonatal; Prenatal in USA	Childhood	Variable
Lifespan	Unimpaired	Death in early adulthood	Variable (unimpaired in types I, and IV), possibly somewhat decreased in type III) [81]

The three specific objectives of this review research were: i) to categorize the types of non-medical impacts of achondroplasia, DMD and OI; ii) to summarize these impacts; and iii) to discuss findings on these impacts across the three diseases.

## Methods

While systematic reviews synthesize the complete nature of a particular field, outlining what approaches are effective and where further research is required, scoping reviews are exploratory projects that map the literature available on a topic, identifying the key concepts, theories, sources of evidence, and gaps in the research [10]. We therefore opted for a scoping review to allow for a quick mapping of the “key concepts underpinning a research area and the main sources and types of evidence available” [11]. We adopted a broad definition of impact that includes consequences of, but also reactions to, a disease. We followed the five stages suggested for a quality scoping review: i) identification of the research question; ii) identification of relevant studies; iii) selection of studies to include in the review; iv) charting of information and data within the included studies; and v) collating, summarizing and reporting results of the review [11].

### Identification of relevant publications

From March to June, 2013, we conducted a search of the literature in three electronic databases: Web of Science, CINAHL, and MEDLINE. The following two string combinations of keywords were used: [Impact, burden, quality of life, living with, coping, adjustment, well-being, quality of life, effects, impacts, responses, reactions, psychosocial] AND [Rare genetic disease/disorder, rare childhood disease, osteogenesis imperfecta, brittle bone disease, Duchenne muscular dystrophy, achondroplasia, chronic illness, musculoskeletal system, physical disorders]. We complemented our search for publications with a systematic screening of the table of contents of specific journals dedicated to rare diseases (e.g. Orphanet Journal of Rare Diseases) and a screening of references of relevant publications. Opinions, commentaries, letters, editorials, and publications without an abstract were immediately initially excluded. The initial search yielded the identification of 845 publications. After removing 8 duplicates, two authors independently selected publications addressing the non-medical impacts of Achondroplasia, DMD and OI, based on the titles and the abstracts. Publications that addressed a rare genetic disease other than the three retained by the research group were excluded from this review, as were publications focussed on the identification of genes. In the case of disagreement among authors, the full-text publications were reviewed by a third senior author. A total of 65 full-text publications remained at this stage of the review.

### Selection of studies to include in the review

**Figure 1:**
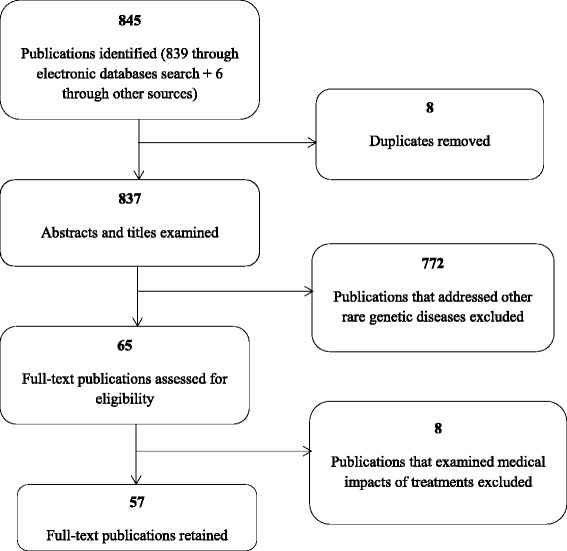
**Study selection flow diagram.**

For inclusion in our study, publications had to be i) published between 1980 and 2013; ii) in English or French; iii) about non-medical effects and responses to OI, achondroplasia and DMD; and iv) have a well-defined methodology. Because a scoping review is not intended to assess the quality of studies, emphasis was not placed on methodological rigor of the retained studies. At this second stage, 8 publications were excluded as they examined medical impacts of a treatment or intervention, and 57 publications were retained. Figure 1 displays the study selection flow diagram. We used a data extraction grid to gather the following items: i) general information (e.g., type of study, country, focus); and ii) the relevance of the article to our study and the scientific information such as concepts, theoretical orientation, methodology, and research tradition.

### Collating, summarizing and reporting results of the review

**Figure 2:**
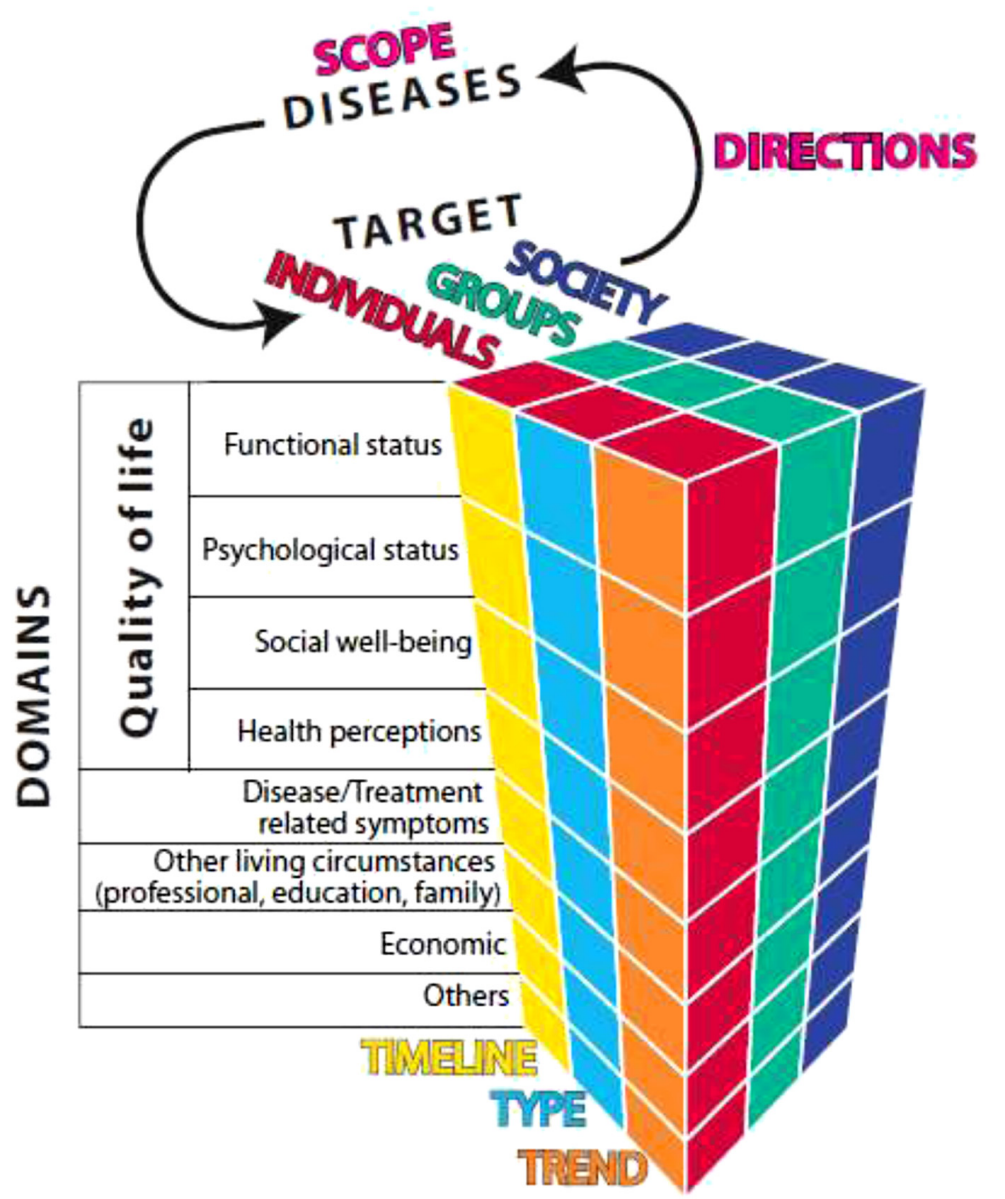
**Framework redefining impacts of rare genetic diseases.**

The 57 retained publications were coded using qualitative structure coding [12] with Nvivo 10 (QSR International) and Endnote X6. From this, we identified main study themes and grouped based on similarity. We iteratively constructed a working framework (Figure 2) to examine separately the extent, range and research activity for OI, achondroplasia and DMD. The adequacy of the framework to review question was tested independently by two authors (MJD and ED) on 10 selected articles. Disagreements were solved by discussion among these authors or by the adjudication of a senior author (FR).

To ensure consistent mapping and analysis of data, we developed a glossary of codes used to classify the impacts as follows: i) ‘scope’ refers to whether the article addresses one or several pediatric rare musculoskeletal disorders; ii) ‘direction’ was used to capture whether the article studied the ‘Effect of Disease’ (Direction 1) or ‘Response to the Disease’ (Direction 2); iii) ‘target’ refers to three distinct groups of focus: ‘individuals’ such as patients, adults, carers, or siblings; ‘groups’ such as physicians, caregivers, or school teachers; and ‘societies’ such as communities or society as a whole; iv) ‘timeline’ and ‘trend’ refer to the temporal analysis of impacts; and v) ‘type’ refers to positive or negative consequences; finally vi) ‘domain’ refers to the aspect of QOL being studied (psychological, functional and so on) or the research tradition or academic discipline used to study the impacts. Once the 57 publications were categorized, we performed a narrative review of the findings on the impacts separately for each disease, and then critically compared the findings across the three diseases. An overview of the distribution of the publications using the components of our working framework is presented, as well as a summary of the major findings of the publications achondroplasia, DMD and OI. Finally, we discuss how research compares across the three diseases.

## Results

A total of 57 publications on non-medical impacts are included in this review study: 3 about achondroplasia [13-15], 39 about DMD [16-54] and 15 about OI [55-69].

### Overview of the publications

The majority of the publications (50) were original research focused on a single disease, while 6 publications concerned two diseases (DMD and other diseases), and 1 publication was a literature review.

The target group assessed was spread between the experiences of the individual with the disease (26), the impact of the disease on families (18), the experience of caregivers only (8), or were focused on the impacts of other groups or tool development and reviews of the literature (5; physicians’ attitudes, the effects on community).

The direction of the impacts was explicitly stated in 44 of the 57: 31 addressed the effects and impacts of living with achondroplasia, DMD or OI, 13 addressed responses to living with the disease on individuals, groups or society (such as resilience, adjustment, coping strategies and attitudes). In these 44 papers, the occurrence of a RGD was conceptualised as a source of stress to which people react. The direction was either a combination of directions, or not explicitly stated, in the remaining 13 papers. Table 2 outlines the studies according to the scope, target, direction, timeline, trend, and type of impacts.

**Table 2:** **Overview of the publications according to diseases, scope, target and directions of the impacts**

**Disease**	**Scope of the impacts**		**Target of the impacts**				**Directions of the impacts**		
	**One disease**	**Multiple diseases**	**Patients only**	**Carers only**	**Families**	**Other groups/society**	**Effects of the disease**	**Responses to the disease**	**NA**
Achondroplasia (N =3)	3, [13-15]	0	2, [13,15]	0	1, [14]	0	2, [13,15]	1, [14]	0
DMD, (N =39)	33, [16,18-20,22-34,38,40-54]	6, [17,21,35-37,39]	15, [20,21,23,29,33,34,41,43,45,47,50,51,53,54]	7, [16,24,26,27,36,39,44]	12, [18,19,23,25,28,31,32,35,46,48,49,52]	5, [17,37,38,40,42]	15, [19,20,22,23,27,31,33,39,41,43,44,50-53]	11, [16,24-26,28-30,34,47-49]	13, [17,18,21,32,35-38,40,42,45,46]
OI, (N = 14)	15, [55-69]	0	9, [55,59,61-64,66,68,69]	1, [65]	5, [56-58,60,67]	0	14, [55-63,65-69]	1, [64]	0

Papers were also spread over several domains (as defined above), with 15 examining the quality of life (QOL) through a normative and validated measure of function, psychosocial well-being, and perception of health (2 achondroplasia, 7 DMD, and 6 OI). Psychosocial impacts on their own were addressed in 18 publications (14 DMD, 4 OI), and functional status on its own was addressed in 10 publications (1 achondroplasia, 6 DMD, 3 OI). The remaining 14 publications assessed the domains of illness experience, utilisation of healthcare services, palliative care, medical staff reactions to the disease, or newborn screening. Tables 3, 4 and 5 report the general information and major findings of the publications for the domain.

**Table 3:** **Summary of findings of publications on non-medical impacts of achondroplasia**

**Publication**	**Major findings**	**Domain**	**Instruments used**	**Sample (N)**	**Country**
Gollust, Thompson et al. [14]	This index measures mean total QOL and indicates higher QOL. Respondents judge their satisfaction with 34 distinct life domains and judge how important each is to their life, producing QOL scores that reflect respondents’ values.	QOL	Survey, Ferrans and Powers Quality of Life Index	189 affected individuals; 136 unaffected 1st degree relatives	USA
Kim, Balce et al. [15]	The physical and functional scores of patients who had surgery were equivalent to those of the nonsurgical group. Even with numerous complications, our data show serial lower limb lengthening is a good option for patients with achondroplasia in terms of good QOL scores. The patients who had surgery for lengthening scored well in the mental component of the SF-36 and in the Rosenberg self-esteem questionnaire.	QOL	American Academy of Orthopaedic Surgeons (AAOS) lower limb, SF-36, Rosenberg self-esteem scores	22 patients who had bilateral leg lengthening, 22 patients who did not	Korea
Ain, Abdullah et al. [13]	Back pain associated with proximal and/or distal leg pain was associated with higher functional disability, more psychological distress, and more physical symptoms. Increase in healthcare use and increase in the number of individuals who had stopped working or changed their type of work because of back or leg pain on follow-up.	Functional limitations and psychological impacts	Symptoms Check List (SCL90R), 10 Beck Depression Inventory (BDI), State-Trait Anxiety Inventory (STAI)	181 affected adults	USA

**Table 4:** **Summary of the findings of publications on non-medical impacts of DMD**

**Publication**	**Major findings**	**Domain**	**Instruments used**	**Sample (N)**	**Country**
Baiardini, Minetti et al. [19]	Using the Children's Health Questionnaire, children with DMD were significantly limited in performing physical activities and demonstrated difficulties with school and other daily activities. The family dynamic appeared to remain intact, but some parents and caregivers reported problems with social aspects of their life.	QOL	Children Health Questionnaire-Parent Form 50, Family Strain Questionnaire	21 mothers, 6 fathers or DMD patients	Italy
Bray, Bundy et al. [22]	Parents of boys with DMD reported significantly poorer health and physical functioning than healthy controls. Parents reported that transitioning to a wheelchair was a particularly difficult time.	QOL	Child Health Questionnaire (CHQ-PF50)	34 boys with DMD, 17 parents at follow up	Australia
Bray, Bundy et al. [23]	Boys with DMB reported significantly lower health-related quality of life compared to the general population, with a moderate to poor correlation with reports from their parents, with parents generally underestimating quality of life of their sons.	QOL	Pediatric Quality of Life Inventory (PedsQLI)	35 parent-son dyads of boys with DMD	Australia
Uzark, King et al. [52]	QOL scores for boys with DMD were significantly lower than those for healthy children for physical and psychosocial QOL. Psychosocial QOL scores were highly impaired particularly in older patients and not significantly associated with use of mobility aids.	QOL	PedsQL 4.0 Generic Core and DMD Module Scales	203 families of boys with DMD	USA
Kohler, Clarenbach et al. [41]	Patients with DMD reported a high level of perceived quality of life. Despite severe limitations in physical functioning, patients reported less role limitations, physical and mental problems, and coping abilities.	QOL	Medical Outcome Questionnaire Short Form 36 (SF-36)	35 male patients with DMD	Switzerland
Manzur, Kinali et al. [42]	There have been improvements in general care and treatment modalities for patients with DMD, with survival into adulthood now a possibility. Multidisciplinary and holistic treatment approaches are also recommended. Curative therapeutic strategies such as cell and gene therapy are currently experimental.	QOL	N/A – Literature Review	N/A	UK
Simon, Resende et al. [50]	Comparing different age groups, patients with DMD did not lose QOL, even with disease progression. In spite of the progressive course of the disease, the QOL in patients with DMD does not worsen.	QOL	Life Satisfaction Index for Adolescents (LSI-A)	95 patients with DMD	Brazil
Garralda, Muntoni et al. [32]	Parents with children with KAFOS reported satisfaction with the rehabilitation process, but some expressed a desire to have had more information beforehand. Families of children with more severe disability reported higher levels of mental distress during the process.	Functional limitations	Life Satisfaction Index for Adolescents (LSI-A)	17 parents and 9 children with DMD	UK
Bendixen, Senesac et al. [20]	When compared with unaffected controls, boys with DMD displayed significantly lower participation in both physical and social activities. Boys with DMD also demonstrated significantly lower QOL scores for physical, social, and school-related domains.	Functional limitations	Semi-structured interviews, Functional Disability Inventory (FDI), The Strengths and Difficulties Questionnaires (SDQ), General Health Questionnaire (GHQ-28), Impact of Events Scale (IES)	50 boys with DMD; 25 unaffected aged-matched controls	USA
Hanayama, Liu et al. [33]	For patients with DMD, commonly observed symptoms associated with dysphagia included oral residuals, coughing while eating, choking while eating, and the need to clear the throat. This demonstrated that a significant portion of patients with DMD had swallowing-related symptoms into their teenage years.	Functional limitations	Children’s Assessment of Participation and Enjoyment, Pediatric Quality of Life Inventory TM 4.0	31 males with DMD	Japan
Marini, Lorusso et al. [43]	The DMD group demonstrated reduced abilities in language processing and cognition, specifically visual attention, but not in receptive or expressive lexical abilities. As well, the narrative speech in subjects with DMD was reduced compared to controls, with shorter sentences.	Functional limitations	Survey of swallowing related symptoms (modified version of previously published measure)	21 males with DMD, 40 healthy controls	Italy
van Wijk, Messelink et al. [53]	Lower urinary tract symptoms in DMD patients are underreported and underdiagnosed; however, the vast majority of male DMD patients with symptoms experience them as a problem, often reducing quality of life.	Functional limitations	Assessment of narrative abilities using the “Nest Story”	199 patients with DMD	Netherlands
Read, Kinali et al. [48]	Unaffected siblings demonstrated comparative psychological functioning and wellbeing scores; however, there was a trend towards increased risk for psychological and emotional problems. Siblings also reported a high impact of their affected sibling's illness on their lives.	Psychological impacts	Strengths and Difficulties Questionnaire (SDQ), General Health Questionnaire (GHQ), Hospital Anxiety and Depression Scale (HADS), SF-36, Functional Disability Inventory (FDI), Family Assessment Device (FAD), Family Burden Interview Schedule and Life Events Checklist	46 unaffected siblings of children with DMD	UK
Read, Kinali et al. [49]	Siblings reported feeling left out when the affected sibling required extra attention, yet the majority remained psychologically well adjusted.	Psychological impacts	Semi-structured interviews and questionnaires previously developed and published by the authors.	35 sibling/parent dyads of children with DMD	UK
Kenneson and Bobo [39]	About half of the caregivers experienced a high level of caregiving demand based on the abbreviated four-item ZBI, consistent with previous report using the full ZBI scale.	Psychological impacts	Survey, components from: Johnson & Johnson Stress Profile, Kessler (K6), Behavioral Risk Factor Surveillance System (BRFSS), Zarit Burden Interview (ZBI), Brief Resilient Coping Scale, ENRICHD Social Support Instrument (ESSI)	1238 Woman caregivers for individuals with DBMD	USA
James, Hadley et al. [35]	Guilt in parents of those with disabilities is associated with depression, helplessness, hopelessness, and disruption of effective parenting. Maternal guilt appears common in XL conditions. Understanding reproductive risks had no significant impact on level of worry among all family members implies that, at least for fathers, siblings, and affected adults, worry is related less to empiric risks and understanding of those risks than to the experience of having an affected family member.	Psychological impacts	Questionnaire developed by authors	112 members of 51 families with chronic granulomatous; 96 members of 51 families with DMD	USA
Hendriksen, Poysky et al. [34]	The results demonstrate the PARS-III is a reliable and valid measure for screening psychosocial adjustment for individuals with DMD. As well, older patients demonstrated higher overall psychosocial adjustment.	Psychological impacts	Personal Adjustment and Role Skills Scale (PARs-III), Revised Rutter Scale	287 parents of boys with DMD	Netherlands, USA
Abi Daoud, Dooley et al. [16]	Parents with a son with DMD were more likely to have an episode of major depression and to have consulted a mental health professional during the last year, and to demonstrate lower self- esteem, than parents in a control group.	Psychological impacts	Depression Scale comprised of items from the World Health Organization's Composite International Diagnostic Interview Short Form (CIDI-SF)	42 parents from 26 families 27 males with DMD	Canada
Chen [24]	Parents of children who were diagnosed at an earlier age reported an increase in ability to cope as a family. Child's level of disability and family hardiness was not correlated to level of family function.	Psychological impacts	Family Hardiness Index, Family Assessment Device, Family APGAR, Duke Health Profile	126 parents of children with DMD	Taiwan
Chen, Chen et al. [25]	Parents with a child with DMD were more aggressive about obtaining resources for their child, with higher stress levels for mothers, and families with a lower income. Coping strategies were used more often in mothers, but overall, fewer coping strategies for emotional expression, self-blame, information seeking, and threat minimization were made.	Psychological impacts	Chronic Impact and Coping Instrument (CICI); Subscales of the Felton 1984 Coping Scale	31 parents of children with DMD, 30 control parents	Taiwan
Chen and Clark [27]	Parents' employment and education, as well as perceived child health and family hardiness/support, was correlated with parental health. Promotion of family hardiness can be supported by nursing interventions and caregiving programs.	Psychological impacts	Family APGAR	126 parents of children with DMD	Taiwan, USA
Garralda, McConachie et al. [31]	Most families reported a positive emotional impact from the trial, with minimal negative impact. Families reported having adequate knowledge and information about the study. Some families reported negative impacts and disappointments during the trial when their child's health deteriorated.	Psychological impacts	Parental Stress and Supports Questionnaire (PSSQ), General Health Questionnaire (GHQ-28), Strengths and Difficulties Questionnaire (SDQ)	19 children with DMD and their families	UK
Chen and Clark [26]	There were significant correlations between age at diagnosis and family function, with better outcomes associated with a younger age at diagnosis. As well, the level of the child's disability was not associated with family function. However, parents did report poorer overall health and an increase in anxiety, depression, and pain and disability, compared to the general population.	Psychological impacts	Barthel Index, Chinese version of Duke Health Profile 27, Family APGAR28, The Family Hardiness Index13, McMaster Family Assessment Device (FAD).	8 single fathers, 26 single mothers, 46 couples with children with DMD	USA
Parsons, Clarke et al. [46]	Prenatal screening for DMD was supported by most families, however anxiety levels for the screened group was higher.	Psychological impacts	Structured question developed by authors, FIRST scores, State Scale, General Health Questionnaire (GHQ)	20 families w/ child w/ DMD, 18 transient, 16 control w/ later diagnosis, 43 control w/ no diagnosis	UK
Cyrulnik, Fee et al. [28]	By parental report, children with DMD demonstrated significant delays in adaptive behaviour skills. Children with DMD also performed more poorly on neuropsychological tests.	Psychological impacts	Vineland Adaptive Behavior Scales, Peabody Picture Vocabulary Test	20 children with DMD, 20 controls	USA
Fee, Hinton et al. [29]	Children living with DMD demonstrated strong behavioural resiliency, which was correlated to high social support and social networks.	Psychological impacts	Child Behavior Checklist (CBCL), Parental Stress Index	146 boys with DMD	USA
Firth, Gardnermedwin et al. [30]	Parents reported problems in three main categories: service, practical, and emotional. Parents were also in favour of prenatal screening and early support, as well as an increase in availability of information. Communication within the family about the disease and implications was also reported as a large area of concern.	Illness experiences	Guided interviews developed by the authors	56 affected boys, 53 families of boys with DMD	USA
Pangalila, van den Bos et al. 2012 [44]	Parents reported that there was substantial burden as caregivers to adults with DMD, specifically surrounding received support, tracheotomy, active coping, and anxiety.	Illness experiences	Caregiver Strain Index (CSI), Self Rated Burden Scale, The EuroQoL-5D, Hospital Anxiety and Depression Scale (HADS), Utrecht Coping List (UCL). General Self-Efficacy Scale	80 parents of 57 adults with DMD	Netherlands
Pehler and Craft-Rosenberg 2009 [47]	The adolescents interviewed did not identify themselves through their diagnosis, and did not see their diagnosis as a crisis. Spirituality and longing was explored as a means to build relationships and connect with others.	Illness experiences	Interview using Manen's Phenomenological method	9 adolescent boys with DMD	USA
Beresford and Sloper 2003 [21]	Participants recognized that they themselves may lack communication skills. The degree of rapport between adolescents and their doctors was influenced by age and gender.	Illness experiences	Semi-structured interviews and group discussion meetings.	63 chronically ill adolescents	UK
Steele, Taylor et al. 2008 [51]	Half of the children have attention deficit–hyperactivity disorder ADHD, which has not been reported in the literature. In the area of internalizing disorders, none of these patients met criteria for depression, either on the KSADS or the self-administered CDI.	Other, assessment of mental health	The Schedule for Affective Disorders and Schizophrenia for School-Age Children (KSADS), Revised Children’s Manifest Anxiety Scale (RCMAS), Children’s Depression Inventory (CDI), Piers-Harris Children’s Self- Concept Scale, Wechsler Intelligence Scale for Children: Third Edition (WISC-III), KSADS-Parent Version, Child Behavior Checklist (CBCL), Conners’ Parent Rating Scale	10 boys with DMD and their parents	Canada
Arias, Andrews et al. 2011 [18]	Fewer than 1 in 5 families were familiar with the term "palliative care", and less than a third had legal plans or advanced directives in place.	Other, use of palliative care	Structured questionnaire developed by authors through focus groups and stakeholder discussions	34 parents of children with DMD	USA
Acharya, Ackerman et al. 2005 [17]	Most physicians support diagnostic genetic testing of high-risk children but are less supportive of expanding newborn screening. Willingness to expand newborn screening does not correlate with professional characteristics but rather with personal interest in testing of their own children.	Other, attitudes of staff	Survey developed by authors	600 paediatricians	USA
Parker, Robb et al. 2005 [45]	While adult patients with DMD deal with a progressive physical impairment due to respiratory, orthopaedic, and other medical factors, areas of disability with often overlooked importance were found in areas of social and medical support.	Other	Retrospective review	25 patients with DMD	UK
Kinali, Manzur et al. 2006 [40]	The survey on the attitudes and practices of UK physicians demonstrated that physicians accept and implement NIV to DMD patients. As well most physicians reported that they promoted shared decision making with DMD patients with respect to NIV. There was also a lack in uniformity of opinion concerning long-term respiratory follow-up for DMD.	Other, attitudes of staff	Modified version of a previously published questionnaire	59 physicians	Canada, UK
Vandervelde, Van den Bergh et al. 2009 [54]	The ACTIVLIM questionnaire showed a good sensitivity to change and could be useful in research settings to characterize the disease course of NMD.	Other	ACTIVLIM Questionnaire	132 patients with NMD	Belgium
Jutai, Rigby et al. 2000 [37]	Our research provides good evidence to support the claim that EADLs (Electronic Aids to Daily Living) contribute significantly to the user's perceived functional independence. Moreover, EADLs appear to enhance other important aspects of the user's psychological well-being, such as feelings of self-confidence and perceived control.	Other, use of electronic device	Psychosocial Impact of Assistive Devices Scale (PIADS)	20 users of EADLs and 21 non-users	Canada
Jarvinen, Lehesjoki et al. 2000 [36]	Carrier testing was in most cases correctly understood and the matter openly discussed. Our results do not suggest that testing in childhood had caused serious harm to the young individuals tested. On the other hand, we found no obvious benefits from this early testing.	Other	Questionnaire developed by authors, RAND 36-item Health Survey 1.0	23 female carries of DMD, 23 females in families with haemophilia	Finland
Kemper and Wake 2007 [38]	Evidence for routine newborn screening for DMD was reviewed, without sufficient data to make a strong recommendation. Further research in the areas of cost, risks, and benefits needs to be conducted.	Other, attitudes towards newborn screening	Literature review		Australia, USA

**Table 5:** **Summary of findings of publications on non-medical impacts of osteogenesis imperfecta**

**Publication**	**Major findings**	**Domain**	**Instruments used**	**Sample (N)**	**Country**
Balkefors, Mattsson et al. [55]	Life satisfaction was high even though health-related quality of life, assessed with the Short Form 36, was significantly lower than the Swedish norm.	QOL	SF-36	29 adults	Sweden
Kok, Uiterwaal et al. [61]	We found little difference in quality of life between the bisphosphonate and placebo groups however in favour of the bisphosphonate group during the two-year follow-up.	QOL	Database review	54 children with OI	Netherlands
Seikaly, Kopanati et al. 2005 [63]	These studies showed a correlation between the severity of the phenotypic expression of OI and QOL indicators tested. Reduced pain, besides being by itself an advantageous outcome, allows effective physical therapy and weight bearing, both benefits that help further strengthen bone. We speculate that improvement in pain resulted in a better sense of well- being and subsequently better self-care scores.	QOL	PEDI, WeeFIM, Bone Mineral Density (BMD)	17 children w/ type III and IV OI	USA
Szczepaniak-Kubat, Kurnatowska et al. 2012 [65]	The child’s disease did not significantly affect either the global quality of life assessment or health of the respondents or their quality of life in terms of physical and psychological status and social relationships. The parents of children with severe OI assessed the life domain associated with the environment they live in as worse than the parents of children with mild OI. The global quality of life assessment of the respondents did not depend on the family’s socioeconomic status or on the help they have been receiving with regard to care for the child.	QOL	WHOQOL-BREF, International Standard Classification of Education (ISCED), Family Affluence Scale (FAS)	25 parents of children with OI	Poland
Widmann, Laplaza et al. 2002 [68]	The results of the SF-36 revealed significantly lower physical function scores compared to the U.S. adult norms. However, the SF-36 mental component scores were equal to the U.S. adult norms. The demographic questionnaire revealed high levels of educational achievement, as well as employment, despite significant physical impairments. The functional questionnaire clearly demonstrated limitations mostly related to ambulation.	QOL	SF-36, Functional Independence Measure (FIM)	30 individuals with OI	USA
Widmann, Bitan et al. 1999 [69]	Physical health as assessed by the SF-36 was closely correlated with vital capacity and scoliosis. Mental health status (assessed by the SF-36 MCS) was not significantly correlated with spinal deformity, chest wall deformity, or pulmonary compromise.	QOL	Questionnaire developed by authors, Short Form-36 (SF-36)	15 patients with OI	USA
Cole 1993 [58]	Psychological aspects studied in a chronologic manner. Impacts on parents, patients, siblings mentioned. Challenges at school, in the society, for the healthcare professionals. Severe, mild and family cases are mentioned.	Psychological impacts	N/A – Opinion paper		Canada
Suskauer, Cintas et al. 2003 [64]	The data suggest that children with OI differ only to the extent that they are relatively hypoactive, and the temperament domains of activity, persistence, and first reaction (approach/withdrawal) may be particularly important for promoting motor achievement.	Psychological impacts	Carey Temperament Scales, Brief Assessment of Motor Function (BAMF), VCOPS, CHAQ, PDH, Pediatric Activity Record (PAR)	35 children w/ OI type III, IV	USA
Bernehall Claesson and Brodin 2002 [56]	Families with children with BBD face many difficulties such as accusations of child abuse, lack of support, and lack of information on the disease. Family activities and family dynamics are also affected by re-occurring fractures and many parents become overprotective.	Illness experience	Questionnaire developed by authors, based on their previous studies, interviews based on the questionnaire	30 families w/ children w/ BBD	Sweden
Brodin 1993 [57]	For children growing up with brittle bones and their families the living conditions are sometimes complicated as they cannot participate in social life together with other young people. Parents of disabled children often complain that their anxieties have been ignored by professionals. No one seems to take them seriously. They have little psychological support and many parents need merely a person to talk to. In order to facilitate habilitation of the child, support should be given to the whole family.	Illness experience	Questionnaires developed by authors and previously published	42 families with children with OI	Sweden
Daly, Wisbeach et al. 1996 [59]	The prognosis for walking in OI was assessed by questionnaire, finding five different patterns of development: abnormal/arrested, delayed/arrested, delayed, and normal. The Sillence and Shapiro classifications were useful in predicting walking. Intramedullary rodding was not advised if the patients had a delay solely in motor development.	Functional limitations	Questionnaire developed by authors	59 families of children with OI	UK
Engelbert, Uiterwaal et al. 2000 [60]	As a predictor of ability to walk in a household, type of OI is the best, along with severity of the collagen defect, and the presence of dentinogenesis imperfecta. Intramedullary rods in the lower extremities have a worse prognosis for walking.	Functional limitations	Questionnaire developed by authors	76 children with OI	Netherlands
Montpetit, Dahan-Oliel et al. 2011 [61]	Young adults with OI type III had significantly lower activity scores in aspects of mobility and domestic life and lower levels of participation in employment, sporting activities and transportation. Participation in leisure and social interactions were not different across OI types.	Functional limitations	Modified questionnaire including scales of the Functional Independence Measure (FIM) and Instrumental Activities Measure (IAM)	54 former OI patients	Canada
Van Brussel, Takken et al. 2008 [67]	A supervised training program can improve aerobic capacity and muscle force and reduces levels of subjective fatigue in children with OI type I and IV in a safe and effective manner.	Functional limitations after physical training	Checklist Individual Strength-20 (CIS-20), Self-perception Profile for Children (CBSK), Child Health Questionnaire Parent-Form 50 (CHQ)	34 children with OI	Netherlands
Tolboom, Cats et al. 2004 [66]	The present study demonstrated a non-significant, but clinically important increase in self-care with a decrease in caregiver assistance regarding self-care after spinal surgery.	Functional limitations after surgery	Harter Self-Perception Profile for Children (SPPC), Paediatric Evaluation of Disability Inventory (PEDI)	11 children with OI	Netherlands

### Narrative review

#### The non-medical impacts of achondroplasia

Evidence from the publications on achondroplasia suggests that affected individuals have an impaired QOL when compared to first-degree relatives [14]. The lower QOL could mainly be linked to psychosocial limitations including lower self-esteem and social stigmatization, to the extent that serial lower limb lengthening appeared a good option to patients despite its numerous complications [13,15]. The functional status of patients with achondroplasia was impaired by back pain and pain in the lower extremity, resulting in some cases in complete cessation of work. However, the functional limitation and psychological distress remained unchanged over time [13].

#### The non-medical impacts of DMD

The overall QOL of parents and their children who are affected with DMD was reported to be lower than in the general population, particularly due to impairments in physical functioning [19,42]. Boys with DMD reported significantly lower QOL than healthy peers in physical and psychosocial domains. Physical limitations increased with age as respiratory problems and muscle weaknesses occurred. However, despite the progressive course of the disease, the psychosocial QOL tended to be higher in adolescents with DMD than in their younger counterparts, suggesting the development of effective coping strategies over time [41,50,52]. For parents, the psychosocial impacts were reported to be great, and quality of life decreased even more around the period of patient transition to wheelchair [22,23]. Finally, parental reports of QOL did not consistently match that of their children, with many underestimating their child’s QOL [23].

The 14 studies that solely examined the psychological impacts of DMD reported higher levels of anxiety, depression, and guilt in parents, particularly in mothers [25,26,46]. However, early diagnosis and family hardiness (the energy resources of the family such as commitment, challenge, and control) were reported to positively influence parental psychological adjustments, the patients’ level of resilience and the reactions of siblings [20,24,26,29,34,43,48,49,51,53].

In addition to the psychological impacts, parents, particularly mothers, reported caring for their children with DMD to be burdensome (help for bathing, toileting), costly in terms of time, and contributing to increased social isolation [44]. Altogether, participation in daily activities and social life was reduced for patients. Siblings of adolescents with DMD seemed more negatively affected than siblings of healthier counterparts.

#### The non-medical impacts of OI

There was little difference in the overall QOL of those diagnosed with OI as compared to the general population. Functional limitations were more important in patients with severe forms of OI, and higher life satisfaction, resilience, low depression and higher social achievements were common [55,64,68]. Parents reported several accusations of child abuse, lack of information on the disease, disruptions of family activities due to the occurrence of fractures, and social isolation [56-58].

### Other findings

Besides these psychosocial impacts of achondroplasia, DMD and OI on families, the impacts on groups and the society were broached in few studies. Two publications examined the attitudes of medical staff about a condition or related treatments options [17,40]. These publications concluded that physicians could promote some experimental treatment options provided that there was a shared-decision making with families. The medical staffs were also found to support newborn and prenatal screening whenever available. Finally, it should be noted that the impacts on the use of health services and economic factors were not assessed in the papers included in this review.

## Discussion

The impacts of achondroplasia, DMD and OI on families reported in this review are derived from in-depth qualitative analysis of subjective illness experiences or objective normative measures of QOL. These methodological specificities resulted in variations in the impacts between and within similar categories of publications that may limit the comparison across the three diseases. However, caring for children particularly with DMD and OI was burdensome because of daily practical problems (bathing, toileting), increased time costs and social isolation, as reported in studies on other RGDs [70-72]. Consequently, carers reported lower QOL particularly in critical periods of loss of ambulation in boys with DMD and during the occurrence of fractures in OI as recently confirmed [73].

As for the QOL in patients with these three diseases, findings seem contradictory with some publications reporting higher QOL and other reporting lower QOL when compared to healthier unaffected controls [20,22]. The severity of functional and physical impairments follow the severity of the disease, and could be seen as increasing from small functional impairment, such as back pain in achondroplasia, to restricted ambulation from bone deformities and numerous fractures in OI, to progressively reduced ambulation and confinement to a wheelchair in DMD [22]. On the contrary and strikingly, the psychological aspect of QOL in patients did not parallel the severity of the disease. For example, DMD is life-threatening and its psychosocial impacts are expected to be more severe than for achondroplasia and OI; however, patients with OI did not seem to experience less psychological distress than those with DMD [41,69]. As for the course of the disease, the stable (unchanged) level of psychological distress in patients with achondroplasia reflects the non-progressive course of the disease [13]. The “up and down” pattern of psychological distress in patients with severe OI also confirms the occurrence of fractures as critical periods over the chronic course of OI [73]. While functional limitations increased, the self-reported psychosocial QOL of boys with DMD did not decrease with age. They may have developed effective coping strategies that ought to have been better documented [52]. This finding was confirmed in previous studies. Indeed the level of impact on family including psychological stress was high for families who had children affected by rare genetic metabolic conditions even when the child’s function was not impaired and the disease was not severe [5].

Some practical implications emerged from this review that can benefit families and caregivers. The correlation between improved parental coping strategies and early diagnosis, better emotional functioning of the child and siblings suggests that there have been efforts to obtaining an early diagnosis and promoting family hardiness for people living with these diseases [74,75]. To that end, interprofessional teams of geneticists, psychologists and social workers in reference centers for these rare diseases could be effective by offering newborn screening, family-centered and life-span psychosocial support whenever possible [76]. Besides, informal support from family and friends but also institutional health and social support such as respite care can contribute to the relief of the burden of care on families.

From the limited number of publications on the impacts of DMD, achondroplasia and OI, we have learned that research about non-medical impacts of achondroplasia, DMD and OI on healthcare systems and society seems very limited and could be further addressed. However, the selection of the keywords used in the searching strategy in this review might have resulted in the exclusion of some publications. For example, challenges in providing adequate updating training to clinicians and staff on these rare diseases as acknowledged in other RGDs could be examined. In addition, research on healthcare-related impacts might compare the use of healthcare resources according to the severity of the disease. Efficient care delivery models organised around primary care whereby family physicians can help to achieve a smooth transition to adult care institutions must also be investigated [56,77,78]. In addition more theoretical understanding is needed about the complex and non-linear interrelationships between two aspects of QOL: the available quality of care and the family functioning and economic status [79-81]. Such understanding will help to acknowledge the relative impacts of these determinants on overall QOL and will help to prioritize adequate interventions.

We acknowledge that methodological limitations could have compromised the inclusion of publications reviewed here. Thus, further longitudinal studies combining normative approaches with qualitative in-depth investigations and comparing several diseases could enhance the understanding of patients’ effective coping strategies. Simultaneous use of parent-report and patient-report questionnaires could provide increased insights [22]. Furthermore, our in-house working framework that guided our organization and analysis of the scoping review could aid others in mapping and organizing the literature during a scoping review; however, to extend its use beyond this would require further validation.

## Conclusion

In general, DMD and OI negatively impacted carers. Some events seem particularly critical such as occurrence of fractures or loss of ambulation in patients. As for patients, functional limitations seemed to follow the severity of the disease, but psychological distress did not, which calls for a better understanding of effective coping strategies. This conclusion is supported by the reported higher life satisfaction in very severely affected patients at different stages of the progressing course of DMD, and the reported resilience characteristic of adolescents with OI. In face of the great difficulties presented to families, life span and family-centered psychological support must be developed in a timely fashion to aid patients, carers, and siblings.

## Abbreviations

DMD: Duchenne muscular dystrophy
OI: Osteogenesis imperfecta
QOL: Quality of life
RGD(s): Rare genetic disease(s)
